# Prevalence of Age-Related Macular Degeneration in Nakuru, Kenya: A Cross-Sectional Population-Based Study

**DOI:** 10.1371/journal.pmed.1001393

**Published:** 2013-02-19

**Authors:** Wanjiku Mathenge, Andrew Bastawrous, Tunde Peto, Irene Leung, Allen Foster, Hannah Kuper

**Affiliations:** 1 International Centre for Eye Health, Department of Clinical Research, London School of Hygiene & Tropical Medicine, London, United Kingdom; 2 Department of Ophthalmology, Kigali Health Institute, Kigali, Rwanda; 3 The Fred Hollows Foundation–Eastern Africa, Nairobi, Kenya; 4 National Institute for Health Research Biomedical Research Centre at Moorfields Eye Hospital NHS Foundation Trust and UCL Institute of Ophthalmology, London, United Kingdom; Kilimanjaro Centre for Community Ophthalmology, Tanzania, United Republic of

## Abstract

**Background:**

Diseases of the posterior segment of the eye, including age-related macular degeneration (AMD), have recently been recognised as the leading or second leading cause of blindness in several African countries. However, prevalence of AMD alone has not been assessed. We hypothesized that AMD is an important cause of visual impairment among elderly people in Nakuru, Kenya, and therefore sought to assess the prevalence and predictors of AMD in a diverse adult Kenyan population.

**Methods and Findings:**

In a population-based cross-sectional survey in the Nakuru District of Kenya, 100 clusters of 50 people 50 y of age or older were selected by probability-proportional-to-size sampling between 26 January 2007 and 11 November 2008. Households within clusters were selected through compact segment sampling.

All participants underwent a standardised interview and comprehensive eye examination, including dilated slit lamp examination by an ophthalmologist and digital retinal photography. Images were graded for the presence and severity of AMD lesions following a modified version of the International Classification and Grading System for Age-Related Maculopathy. Comparison was made between slit lamp biomicroscopy (SLB) and photographic grading.

Of 4,381 participants, fundus photographs were gradable for 3,304 persons (75.4%), and SLB was completed for 4,312 (98%). Early and late AMD prevalence were 11.2% and 1.2%, respectively, among participants graded on images. Prevalence of AMD by SLB was 6.7% and 0.7% for early and late AMD, respectively. SLB underdiagnosed AMD relative to photographic grading by a factor of 1.7.

After controlling for age, women had a higher prevalence of early AMD than men (odds ratio 1.5; 95% CI, 1.2–1.9). Overall prevalence rose significantly with each decade of age. We estimate that, in Kenya, 283,900 to 362,800 people 50 y and older have early AMD and 25,200 to 50,500 have late AMD, based on population estimates in 2007.

**Conclusions:**

AMD is an important cause of visual impairment and blindness in Kenya. Greater availability of low vision services and ophthalmologist training in diagnosis and treatment of AMD would be appropriate next steps.

*Please see later in the article for the Editors' Summary*

## Abstract 2

Using digital retinal photography and slit lamp examination in a population-based sample in the Nakuru District of Kenya, Andrew Bastawrous and colleagues determined the prevalence of age-related macular degeneration in adults 50 years and older.

## Editors' Summary

**Background:**

Worldwide, 39 million people are blind, and 246 million people (mainly living in developing countries) have moderate or severe visual impairment. The third leading global cause of blindness (after cataracts and glaucoma) is age-related macular degeneration (AMD). This group of conditions is characterized by lesions in the macular (central) region of the retina, the tissue at the back of the eye that converts light into electrical messages and sends them to the brain. AMD, which affects older people, destroys the sharp central vision that is needed for reading or driving, leaving only dim, blurred images or a black hole at the center of vision. AMD can be diagnosed by examining digital photographs of the retina or by examining the retina directly using a special magnifying lens (slit lamp biomicroscopy). There is no cure for AMD, although injections into the eye of certain drugs, such as bevacizumab, that block the activity of vascular endothelial growth factor can slow the rate of vision loss caused by some forms of AMD.

**Why Was This Study Done?:**

Most investigations of the prevalence (the proportion of a population with a disease) of AMD and of risk factors for AMD have studied people with European or Asian ancestry. Very little is known about AMD in African populations, and the data that are available mainly come from African populations living outside Africa. It is important to know whether AMD is an important cause of visual impairment and blindness in Africa, so that informed decisions can be made about the need for AMD programs in African countries. In this cross-sectional population-based study, the researchers investigate the prevalence of AMD among people aged 50 years or older living in Nakuru District (an ethnically diverse region of Kenya) and look for predictors of AMD in this population. In a cross-sectional population-based study, researchers observe a representative subset of a population at a single time point.

**What Did the Researchers Do and Find?:**

The researchers randomly selected 100 clusters of 50 people aged 50 years or older for their study. Between January 2007 and November 2008, study participants had a comprehensive eye examination and completed a standardized interview that included questions about their age, gender, other demographic details, medical history, and exposure to possible risk factors for AMD. Based on digital retinal images, the prevalences of early and late AMD among the study population were 11.2% and 1.2%, respectively. The prevalences of early and late AMD judged by slit lamp biomicroscopy were 6.7% and 0.7%, respectively. After controlling for age, women had a higher prevalence of both early and late AMD than men. The overall prevalence of AMD rose with age: compared to the youngest age group, the oldest age group had a three-fold higher risk of developing late AMD. Of the people with any grade of AMD, 25.6% had some visual impairment and 2.5% were blind. Overall, 9.9% of the blindness seen in the study was attributable to AMD.

**What Do These Findings Mean?:**

These findings identify AMD as an important cause of visual impairment and blindness in Nakuru District, Kenya. Extrapolation of these findings to the whole of Kenya suggests that 283,900 to 362,800 Kenyans had early AMD and 25,200 to 50,500 had late AMD in 2007. The accuracy of these findings is limited by the inability to obtain digital retinal images from all the participants (often because of electricity failures) and by other aspects of the study design. Moreover, because the methodology used in this study differed from some other studies of AMD, the prevalence of AMD reported here cannot be compared directly to those found in other studies. Nevertheless, these findings have several important implications. In particular, although recent evidence suggests that bevacizumab is likely to be both effective and affordable in Africa, the infrastructure required to deliver an adequate AMD service is currently prohibitively expensive in most African countries. Thus, these findings suggest that it is essential that research is undertaken to support the development of AMD treatment programs that are affordable and deliverable in Africa, and that low vision resources are provided for individuals with vision impairment.

**Additional Information:**

Please access these websites via the online version of this summary at http://dx.doi.org/10.1371/journal.pmed.1001393.

- The US National Eye Institute provides detailed information about age-related macular degeneration
- The UK National Health Service Choices website also provides information about age-related macular degeneration, including personal stories about the condition
- The UK Royal National Institute of Blind People has information on age-related macular degeneration, including a video of a person describing their experiences of the condition
- AMD Alliance International provides written and audio information in several languages about age-related macular degeneration, including a large selection of personal stories; the Macular Degeneration Partnership also provides information about age-related macular degeneration, including a simulation of the condition
- MedlinePlus has links to additional resources about age-related macular degeneration (in English and Spanish)

## Introduction

In the latest estimates of global blindness and visual impairment undertaken by the World Health Organization, in 2010, age-related macular degeneration (AMD) is the third most common cause of blindness worldwide behind cataracts and glaucoma [1]. It has remained an important cause of blindness globally since the last World Health Organization survey in 2002, in which it was identified as the leading cause of blindness in high-income countries [2]. As the global population ages, AMD is likely to increase in importance. Currently, no curative treatment exists. The recent promise of anti–vascular endothelial growth factor treatments is unlikely to offset the growth of AMD globally, as these treatments are only useful in exudative AMD and are not currently widely accessible outside of high-income countries. Nutritional interventions can reduce the progression of certain subtypes of early AMD [3]; however, protective levels of required vitamins and minerals are difficult to obtain in a healthy diet, and the cost of supplementation is prohibitive for many who could potentially benefit [4].

The majority of data on AMD available globally is from population-based studies undertaken in white and Asian populations [5]–[13], and few data are from peoples of African descent. The data that do exist for individuals of African descent are largely from studies undertaken in populations living outside of the African continent [14],[15]. It is presumed that AMD is rare in Africans; however, in the last 10 y, African population-based studies have suggested that posterior segment eye diseases are highly prevalent, and this group of disorders, which includes AMD, diabetic retinopathy, and glaucoma, has been highlighted as either the leading or second leading cause of blindness in surveys undertaken in Cameroon [16], Tanzania [17], Kenya [18], Rwanda [19], Zanzibar [20], and Guinea [21]. These studies, however, did not assess AMD as a specific entity and did not use digital retinal photography. Moreover, comparative data for whites and Africans living in the same geographical area (Baltimore, Maryland, US) have suggested differing predispositions towards AMD, with possible genetically protective factors for AMD progression in individuals of African descent compared to their white counterparts [22]. Population-based evidence for African populations living in Africa on the prevalence of the disease, and of the risk factors for AMD, is therefore important.

The purpose of this study was to estimate the prevalence and risk factors for AMD in the age group 50 y and over in an African population in Nakuru District, Kenya, using digital retinal photography and slit lamp biomicroscopy (SLB). Nakuru District is within the Rift Valley Province in Kenya, with a population of nearly 10 million, approximately one-quarter of the Kenyan population. Nakuru is diverse in its ethnic mix (all 42 tribes present in Kenya represented within the district), range of socioeconomic activity, and urban/rural mix.

## Methods

### Ethics Statement

Ethical approval was granted by the London School of Hygiene & Tropical Medicine Ethics Committee and by the Kenya Medical Research Institute. Approval was also granted by the Rift Valley Provincial Medical Officer and the Nakuru District Medical Officer of Health. Written approval was sought from the administrative head in each cluster, usually the village chief. All participants gave written or verbal consent to participate. People requiring medical treatment were referred to the appropriate centre.

### Sampling Strategy and Recruitment

The study fieldwork was carried out in two phases, from 11 January to 2 June 2007 and from 8 April to 11 November 2008.

Recent census data for Kenya were not available [23], and therefore election roll lists that were renewed in 2006 in preparation for the 2007 general elections were used as the sampling frame for this survey. 100 clusters were selected with a probability proportional to the size of the population. A cluster was defined as the area served by a polling station.

Households were selected within clusters using a modified compact segment sampling method [24]. Each cluster was divided into segments so that each segment included approximately 50 people aged ≥50 y. One segment was selected at random, and all eligible people were included sequentially until 50 had been examined. Location data including GPS coordinates of houses and mobile phone contacts were taken to allow follow-up of all those examined.

This sample size was sufficient to estimate a prevalence of AMD of 3.0% among those aged 50+ y, with a required precision of 0.5%, 95% confidence, a design effect to account for clustering of 1.5, and a response rate of 90% (Epi Info 6.04, US Centers for Disease Control and Prevention).

### Ophthalmic and General Examination

Suitable predetermined examination sites were selected, on the recommendation of the village leader, with close proximity to the cluster and to electricity supply (mains or generator).

### Visual Acuity

The presenting visual acuity was defined as the number of letters read correctly without glasses if the participant did not have glasses or with glasses if they had them. Testing was done on each eye separately at 4 m using a reduced LogMAR tumbling “E” chart [25] in a well-illuminated area, as described elsewhere [26].

### Fundus Photography

The participants had two nonstereoscopic digital 45° fundus photographs taken per eye by an ophthalmic clinical officer using a TRC-NW6S Non-Mydriatic Retinal Camera with a ten-megapixel Nikon D80 camera (Topcon). One image was centred on the optic disc, while the other was centred on the macula. The digital images were stored on hard disc, backed up on an external drive, and one copy saved on CD was forwarded to the Retinal Grading Centre at Moorfields Eye Hospital Reading Centre in London for grading and confirming the clinical diagnosis of posterior segment disease.

### Image Grading

The senior grader (I. L.) graded all images. All images were first categorised for quality as excellent, good, fair, borderline, or ungradable. All questionable lesions and all eyes classified as having late-stage AMD were adjudicated by the Moorfields Eye Hospital Reading Centre clinician (T. P.). Any lesions considered to be due to other causes such as myopia and inflammatory disease were not graded for AMD, and these were also verified by T. P. The adjudicator also graded 5% of randomly selected images to ensure quality control. Data were entered into Excel and checked for consistency by a data monitor. Those with images were classified as “image group” for further analysis.

### Retinal Examination

SLB was performed after pharmacological dilatation with guttae tropicamide 1% using a 90 diopter lens. Assessment was inclusive of the macula, the retinal vasculature, and the peripheral retina. The view of the retina was recorded as clear, hazy, or no view. The macula was examined for presence of drusen, hypo- or hyper-pigmentation, dry AMD or geographic atrophy (GA), and neovascular changes. Any other pathologies of the retina or vitreous, e.g., retinal detachments or vitreous haemorrhages, were also noted, and a description given. Those with slit lamp examination were classified as “SLB group”.

### Definitions Used

A modified version of the International Classification and Grading System for Age-Related Maculopathy and Age-Related Macular Degeneration [27] was used for image grading. Drusen were categorised based on size, uniformity of colour, and margins. Based on these, patients were classified into hard or soft drusen categories. Small drusen, less than 63 µm, were considered to be hard. Large drusen with a uniform density, sharp margins, and a nodular surface texture were placed in the soft distinct category, whereas those without sharp margins were classified as indistinct. Where end-stage disease was apparent, patients were classified as having geographic atrophy if there were well-demarcated regions with diameters in excess of 175 µm, within which large choridal vessels were clearly visible, owing to the atrophy of the overlying choriocapillaris and retinal pigment epithelium. Neovascular AMD was graded as present when exudative features, such as serous fluid, haemorrhage, lipid exudates, or fibrosis, were seen to be originating primarily from the subretinal and pigmentepithelial tissue layers.

SLB grading of AMD was as follows: (1) drusen present: presence of discrete whitish-yellow spots at the macula area; (2) pigmentary changes present: presence of increased pigment or hyperpigmentation or sharply demarcated areas of depigmentation or hypopigmentation of the retinal pigment epithelium; (3) dry AMD or geographic atrophy: atrophy of the retinal pigment epithelium, with visible underlying choroidal vessels; (4) wet or neovascular AMD: presence of retinal pigment epithelium detachment, subretinal or subpigment epithelial neovascularization, or fibrous scar tissue, haemorrhages, or exudates; (5) no AMD: none of the features described above were present; (6) cannot assess: the retina could not be adequately visualised for grading. Case definitions were based on the eye with more severe status if both eyes were gradable, and on the gradable eye if only one eye was gradable (*n* = 37).

Detailed interviews were undertaken in the local language covering demographic details, information on risk factors, socioeconomic status (SES), and full past medical history. SES was evaluated using a continuous asset score that was produced for each participant using a scoring system derived through principal component analysis in an earlier study in this setting [28],[29]. The scale included assessment of 17 context-specific asset items owned by the household, including different types of furniture, electrical equipment, cattle, and vehicles. Information was collected on five household characteristics, including the building material of the floor, roof, and walls; type of toilet; and the number of rooms. The score was divided into quartiles based on the distribution across all the study participants, to derive a measure of relative SES.

Weight, height, waist and hip circumference, blood pressure, and random cholesterol and glucose blood levels were also measured. “Mother tongue” was used as a measure of tribal affiliation.

### Data Handling and Statistical Analysis Methods

#### Data entry

Data were double-entered into a specially developed dataset (EpiData Entry, version 2.1). Consistency checks were performed each evening, and inconsistencies corrected the same day.

#### Data analysis

The prevalence of AMD was estimated, and the “svy” command in Stata was used with a design effect of 1.5 in order to take into account the cluster sampling survey methodology when calculating confidence intervals around the prevalence estimates.

Statistical analyses were undertaken using Stata. Logistic regression analyses were produced to assess the univariate associations between potential risk factors (age, gender, SES, tribal origin, hypertension, diabetes, angina, cholesterol level, body mass index, waist∶hip ratio, previous cataract surgery, smoking and alcohol consumption, education, and urban versus rural) and prevalent AMD. Multivariable logistic regression models were developed through stepwise selection, with variables retained at the *p*<0.05 level. These analyses were restricted to cases defined from the image group data, since definite disease status was available only for this group.

The data for individuals with both SLB and retinal images were used to calculate a correction factor (if needed) to apply to the SLB group to estimate the overall prevalence of AMD for Nakuru.

Estimated numbers of individuals within Kenya with AMD were extrapolated from population data from the US Census Bureau International Data Base (http://www.census.gov/population/international/data/idb/country.php) by applying age- and sex-specific prevalence estimates for the Kenyan population.

## Results

There were 5,010 eligible individuals identified for this study. Of these, 4,414 participants underwent examination, giving a response rate of 88.1%, and 4,381 had full ophthalmic examination; 33 participants were not included in the ophthalmic analysis because of missing data as a consequence of equipment failure. Of the non-respondents, 584 (98%) were away working or visiting family outside the cluster location, and 12 (2%) refused to participate; none were excluded as a result of inability to communicate. The socio-demographic characteristics of the participant group are described in earlier publications [30]. Out of the 4,381 individuals who had ophthalmic examinations in the Nakuru study, 4,312 (98.4%) were successfully screened for AMD by SLB of the retina (SLB group).

**Figure 1:**
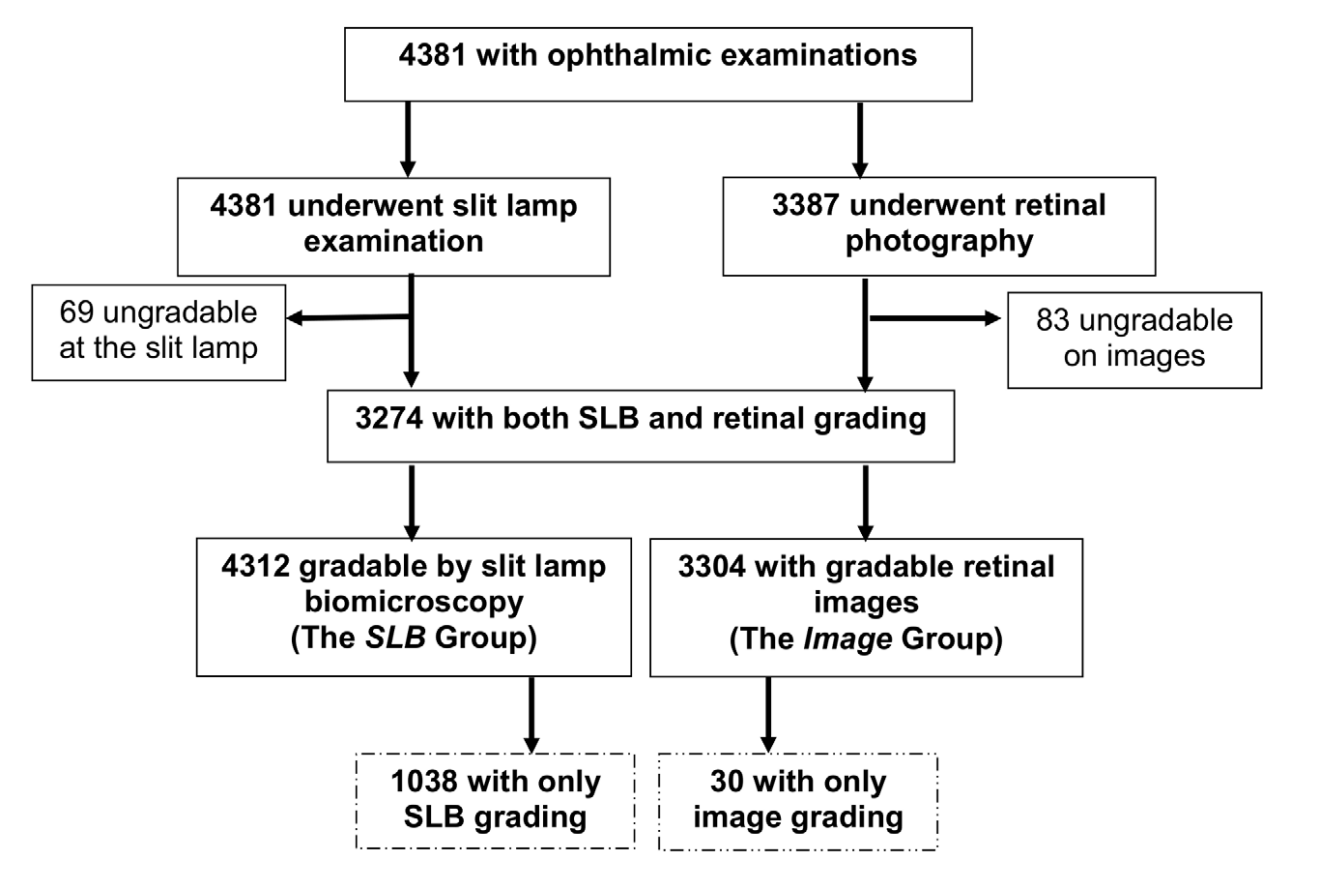
AMD study participation chart.

3,387 (77.3%) participants underwent retinal photography. An image for grading for AMD in at least one eye was available for 3,304 individuals (75.4%) (image group). 3,274 individuals (74.7%) had both methods of screening (Figure 1).

Compared to people in the SLB group, those who had images taken were more likely to be male, younger, urban residents, not Kikuyu, not diabetic, and not visually impaired (Tables 1 and S1). A correction factor was necessary because there were significant differences in the characteristics of the SLB-only group compared to the image group, and so it was not possible to generalise the results from the image group to the whole population.

**Table 1:** Demographic characteristics of study participants (*n* = 4,381).

Attribute	Number (Percent) of Those with Grading by Retinal Images, *n* = 3,304	Number (Percent of Those with Only SLB Diagnoses, *n* = 1,038	Age- and Sex-Adjusted Odds Ratio (95% CI)
**Gender**			
Male	1,629 (49%)	450 (43%)	Baseline
Female	1,675 (51%)	588 (57%)	0.8 (0.7–0.9)
**Age**			
50–59 y	1,508 (78.0%)	426 (22.0%)	Baseline
60–69 y	997 (77.3%)	292 (22.7%)	1.0 (0.8–1.2)
70–79 y	547 (75.8%)	175 (24.2%)	0.9 (0.8–1.2)
80+ y	252 (63.5%)	145 (36.5%)	0.6 (0.4–0.7)
**Environment**			
Rural	2,143 (69%)	774 (75%)	Baseline
Urban	1,161 (31%)	264 (25%)	1.5 (1.3–1.7)
**SES**			
Poorest	783 (24%)	287 (27%)	Baseline
2nd quartile	815 (25 %)	267 (26%)	1.0 (0.9–1.2)
3rd quartile	840 (26%)	243 (23%)	1.1 (0.9–1.4)
Least poor	829 (25%)	252 (24%)	1.1 (0.9–1.3)
**Tribe**			
Kikuyu	1,997 (60%)	721 (70%)	Baseline
Kalenjin	780 (24%)	211 (20%)	1.3 (1.1–1.6)
Other	527 (16%)	106 (10%)	1.7 (1.3–2.1)
**Diabetes**			
Non-diabetic	3,091 (94%)	947 (92%)	Baseline
Diabetic	192 (6%)	86 (8%)	0.7 (0.5–0.9)
**Visual impairment**			
≥6/12	2,891 (88%)	820 (79%)	Baseline
<6/12	3,973 (12%)	218 (21%)	0.5 (0.4–0.6)

The prevalences of drusen and pigmentary irregularities as observed on the fundus images, by gender and age, are shown in Table 2. Note that varying features of AMD, e.g., drusen and pigmentation, can co-exist in a single eye and in both eyes and therefore one individual may be listed under more than one AMD characteristic. However, the prevalence of AMD in the population is based on the grading of AMD in a person. Drusen were present in a large proportion of the population. The most common type of drusen encountered in all ages was small, hard drusen, <63 µm, which were present in 59.1% of the study population. Large, soft drusen, which are considered to be indicative of early AMD, were present in 9.4% of the population. There were significant age trends (X^2^ trend test, *p*<0.001), with increased prevalence of all drusen and all pigmentary changes from age 50 y to age 79 y. The gender difference was less strong, though drusen and pigmentary changes were more common in women than men. The overall prevalence of retinal pigment abnormalities was 4.8% (95% CI, 3.7–6.1). Increased pigment was seen more frequently than depigmentation in all age groups, and prevalence increased from 1.6% in the lowest age groups to 7.2% in those aged 80 y or more. The difference in prevalence of pigment in men and women was not significant (*p* = 0.66).

**Table 2:** Number and prevalence of features of early AMD (image group) and age-related maculopathy by sex and age (*n* = 3,304).

Attribute	Small Drusen (<63 µm)		Large Drusen (≥63 µm)		Hypopigmentation		Hyperpigmentation	
	*N*	Percent (95% CI)	*N*	Percent (95% CI)	*N*	Percent (95% CI)	*N*	Percent (95% CI)
**Total**	1,954	59.1 (56.1–62.1)	310	9.4 (8.2–10.7)	79	2.4 (1.7–3.3)	117	3.5 (2.7–4.6)
**Age**								
50–59 y	813	53.9 (50.0–57.8)	52	3.5 (2.6–4.6)	31	2.1 (1.2–3.4)	24	1.6 (0.9–2.7)
60–69 y	603	60.4 (56.4–64.3)	92	9.2 (7.5–11.4)	26	2.6 (1.7–4.1)	41	4.1 (2.8–6.0)
70–79 y	372	68.0 (63.6–72.2)	103	18.8 (15.4–22.9)	17	3.1 (1.8–5.4)	34	6.2 (4.3–8.9)
80+ y	166	66.1 (59.0–72.6)	63	25.1 (20.1–30.9)	5	2.0 (0.7–5.3)	18	7.2 (4.2–12.1)
***p*** **-Value** a	<0.001	<0.001	<0.001	<0.001				
**Gender**								
Male	889	54.6 (51.0–58.1)	123	7.6 (6.4–8.9)	36	2.2 (1.5–3.2)	45	2.8 (2.0–3.9)
Female	1,065	63.6 (60.0–67.1)	187	11.2 (9.3–13.4)	43	2.6 (1.7–3.9)	72	4.3 (3.1–5.9)
***p*** **-Value** b	<0.001	0.002	0.74	0.05				

a X^2^ trend test for age groups.

b X^2^ trend for sex differences.

Neovascular AMD was more common (0.9%) than geographic atrophy (0.5%) (Table 3). There were significant age trends for both, with geographic atrophy being prevalent in only 0.3% of those in their 50 s and increasing to 2.0% in those age 80 y and over.

**Table 3:** Number and prevalence of features of late AMD (image group) by sex and age (*n* = 3,304).

Attribute	Neovascular AMD		GA		No Late AMD	
	*N*	Percent (95% CI)	*N*	Percent (95% CI)	*N*	Percent (95% CI)
**Total**	29	0.9 (0.5–1.4)	18	0.5 (0.3–0.9)	3,266	98.9 (98.3–99.2)
**Age**						
50–59 y	5	0.3 (0.1–0.9)	3	0.2 (0.1–0.6)	1,503	99.5(99.0–99.8)
60–69 y	10	1.0 (0.5–2.0)	4	0.4 (0.2–1.1)	987	98.8 (97.8–99.8)
70–79 y	7	1.3 (0.6–2.9)	6	1.1 (0.4–2.8)	540	98.2 (96.3–99.1)
80+ y	7	2.1 (1.1–6.8)	5	2.1 (0.8–4.6)	246	96.4 (92.5–98.3)
***p*** **-Value** a	<0.001	<0.001	<0.001			
**Gender**						
Male	10	0.6 (0.3–1.2)	10	0.6 (0.3–1.2)	1,614	99.1 (95.7–97.5)
Female	19	1.1 (0.7–1.9)	8	0.5 (0.2–0.9)	1,652	98.6 (95.2–97.0)
***p*** **-Value** a	0.70	0.80	0.30			

a The features of late AMD, i.e., neovascular changes and geographic atrophy, are not mutually exclusive, so individuals may appear in both columns. The data for late AMD, and hence for “no late AMD”, is person-specific and therefore mutually exclusive. Those with both GA and neovascular AMD are counted in the neovascular AMD group.

The prevalence of all stages of AMD was lower when SLB grading was used than when grading was from retinal images (Table 4). The total prevalence of AMD from SLB was 7.4% (early AMD, 6.7%, and late AMD, 0.7%), while the prevalence of AMD by retinal image grading was 12.4% (early AMD, 11.2%, and late AMD, 1.2%). A smaller proportion of participants were classified as ungradable by SLB (30; 1.6%) than by retinal image grading (83; 2.6%). A correction factor of 1.7 for total AMD needs to be applied for those who did not have retinal imaging (considered the gold standard) to get the true prevalence estimate of AMD in the SLB group.

**Table 4:** Comparison of SLB and image groups.

Variable	Prevalence in Image Group	Prevalence in Same People by SLB	Underdiagnosis Factor for SLB
Number gradable	3,304	3,274	
Early AMD	366 (11.1%)	219 (6.7%)	1.7
Late AMD	38 (1.2%)	24 (0.7%)	1.6
Total AMD	404 (12.2%)	243 (7.4%)	1.7

Data are given as number (percentage).

Sensitivity and specificity analysis for the detection of early AMD and late AMD by SLB grading versus retinal imaging, in those individuals who had both SLB and image grading available, showed that SLB grading had poor sensitivity (early, 21.3%, and late, 36.8%) and good specificity (early, 95.2%, and late, 99.9%).

A total of 404 (366 early AMD and 38 late AMD) cases were confirmed by images, while another 85 cases (75 early AMD and ten late AMD) were detected in the group that received SLB only. Combining all cases gives a total of 489 cases, or a prevalence of 11.3% for AMD (435 [10.2%] early AMD and 48 [1.1%] late AMD) in this population. However, since SLB underdiagnosed AMD by a factor of 1.7, if this factor is applied to the SLB prevalences, an adjusted prevalence of 12.7% (11.4% for early AMD and 1.3% for late AMD) is reached (Table 5).

**Table 5:** Determination of all AMD cases and adjusted prevalence including diagnosis by SLB.

Terms	Early AMD	Late AMD	Total AMD	No AMD
Confirmed by retinal images (*A*) (*n* = 3,304)	366	38	404	2,900
Confirmed only by SLB (*B*) (*n* = 4,312)	75	10	85	953
Total observed cases (*n* = 4,342)	441	48	489	3853
Prevalence	10.2%	1.1%	11.3%	88.7%
95% CI	(9.0–11.5)	(0.8–1.6)	(10.0–12.7)	(87.3–90.0)
Corrected prevalence (*A*+[*B*×1.7 or 1.6])/3,797	11.4%	1.3%	12.7%	87.4%

When extrapolating these data to the entire Kenyan population based on data from 2007, we estimated that there are 283,900 to 362,800 people over 50 y in Kenya with early AMD and 25,200 to 50,500 with late AMD.

Age/sex-adjusted analyses show that only age and gender were significantly associated with early AMD, with those affected more likely to be female and with prevalence increasing with each decade of age (Table 6).

**Table 6:** Risk factors for early AMD.

Category	Risk Factor	Number with Early AMD	Prevalence (95% CI)	Age- and Sex-Adjusted Odds Ratio (95% CI)
**Demographic factors**	**Age group (years)**			
	50–59	82	5.5% (4.2–7.1)	Reference
	60–69	115	11.7% (9.8–13.9)	2.3 (1.7–3.1)
	70–79	110	20.5% (16.7–24.9)	4.7 (3.5–6.4)
	≥80	59	24.3% (19.3–30.1)	5.7 (3.9–8.3)
	**Gender**			
	Female	212	9.5% (7.7–10.6)	1.5 (1.2–1.9)
	Male	154	12.8% (10.6–15.5)	Reference
	**Tribe**			
	Kikuyu	244	12.3% (10.3–14.8)	Reference
	Kalenjin	80	10.4% (8.5–12.8)	0.8 (0.6–1.1)
	Other	42	7.8% (5.8–11.2)	0.9 (0.7–1.3)
	**SES**			
	1st quartile (poorest)	112	14.6% (12.2–17.4)	Reference
	2nd quartile	102	12.6% (10.0–15.8)	1.0 (0.7–1.3)
	3rd quartile	84	10.1% (8.0–12.7)	0.9 (0.7–1.2)
	4th quartile (richest)	65	7.9% (5.8–10.8)	0.8 (0.5–1.1)
**Systemic factors**	**Body mass index**			
	<25 kg/m2	252	12.2% (10.6–14.0)	Reference
	Overweight (25–29.9 kg/m2)	78	10.5% (8.3–13.1)	1.0 (0.7–1.3)
	Obese (≥30.0 kg/m2)	33	7.8% (5.2–11.4)	0.7(0.5–1.0)
	**Diabetes**			
	Yes	15	7.9% (4.3–14.1)	0.7 (0.4–1.1)
	No	350	11.5% (9.9–13.2)	Reference
	**Hypertension**			
	Hypertensive	213	12.8% (10.7–15.2)	1.2 (1.0–1.5)
	Non-hypertensive	152	9.7% (8.2–11.4)	Reference
	**Angina grade**			
	None	290	10.8% (8.7–11.9)	Reference
	Grade 1	55	12.4% (9.3–15.2)	1.2 (0.9–1.7)
	Grade 2	20	16.4% (11.5–22.8)	1.7 (1.0–2.9)
	**Smoking**			
	Current	21	7.8% (5.3–11.2)	0.8 (0.5–1.4)
	Former	77	10.5% (8.5–12.9)	1.0 (0.7–1.4)
	Never	267	11.9% (10.1–14.0)	Reference
	**Alcohol**			
	Current	68	11.6% (9.0–14.7)	1.3 (0.9–1.9)
	Former	160	11.3% (9.6–13.2)	1.0 (0.8–1.3)
	Never	137	11.1% (8.7–14.1)	Reference
**Ocular features**	**Cataract surgery**			
	Yes	26	15.2% (10.6–21.3)	0.9 (0.5–1.3)
	No	339	11.0% (9.5–12.7)	Reference

Modelling age as a continuous variable did not alter the findings (Table S2).

Age/sex-adjusted analyses showed that only age was significantly associated with late AMD, with increased late AMD prevalence with every decade after 50 y (Table 7). All other variables showed no association.

**Table 7:** Risk factors for late AMD.

Category	Risk Factor	Number with Late AMD	Prevalence (95% CI)	Age- and Sex- Adjusted Odds Ratio (95% CI)
**Demographic factors**	**Age group (years)**			
	50–59	7	0.5% (0.2–1.0)	Reference
	60–69	12	1.2% (0.7–2.2)	2.8 (1.1–7.3)
	70–79	10	1.8% (0.9–3.7)	5.1 (1.9–13.5)
	≥80	9	3.6% (1.7–7.5)	10.4 (3.8–28.2)
	**Gender**			
	Female	23	1.0% (0.6–1.7)	1.8 (0.9–3.4)
	Male	15	1.6% (1.0–2.4)	Reference
	**Tribe**			
	Kikuyu	20	1.1% (0.6–2.1)	Reference
	Kalenjin	11	1.5% (0.8–3.1)	1.4 (0.7–3.0)
	Other	7	1.4% (0.7–3.2)	2.2 (0.9–5.5)
	**Environment**			
	Rural	31	1.6% (1.0–2.6)	Reference
	Urban	7	0.7% (0.3–1.45)	0.6 (0.2–1.3)
**Systemic factors**	**Cholesterol**			
	Low	31	1.1% (0.7–1.8)	Reference
	High	4	3.7% (1.4–9.6)	3.0 (1.0–8.9)
	**Smoking**			
	Current	4	1.6% (0.6–4.1)	2.2 (0.7–6.6)
	Former	6	0.9% (0.4–2.0)	1.0 (0.4–2.7)
	Never	28	1.4% (0.9–2.1)	Reference
	**Alcohol**			
	Current	7	1.3% (0.6–3.0)	0.9 (0.3–2.4)
	Former	12	0.9% (0.5–1.8)	0.5 (0.2–1.1)
	Never	19	1.7% (1.1–2.7)	Reference
**Ocular features**	**Cataract surgery**			
	Yes	6	4.0% (1.9–8.1)	2.1 (0.8–5.2)
	No	32	1.2% (0.7–1.8)	Reference

Of the 487 people with any grade of AMD (diagnosed by SLB or retinal images), a total of 137 (28.1%) were visually impaired, including 12 blind people (2.5%; 95% CI, 1.3–4.8), four with severe visual impairment (0.8%; 0.3–2.2), and 82 with moderate visual impairment (16.8%; 13.4–20.9). 350 (71.9%; 7.0–76.4) of those with AMD had normal vision (Table 8). Among the 669 people with visual impairment in the entire Nakuru study, 137 (20.5%) had features of AMD, either exclusively or in combination with other pathology.

**Table 8:** Visual acuity in those with AMD.

Visual Acuity	All AMD from Retinal Images	All AMD from SLB Only	Total AMD	No AMD	Total
**Normal (≥6/12)**					
Number	302	48	350	3362	3,712
Percent	75.1%	56.5%	71.9%	86.3%	84.7%
95% CI	(70.1–79.5)	(44.3–67.9)	(67.0–76.4)	(84.6–87.9)	(82.9–86.4)
**Mild VI (<6/12–6/18)**					
Number	23	16	39	185	224
Percent	5.7%	18.8%	8.0%	4.8%	5.1%
95% CI	(3.9–8.3)	(12.5–27.1)	(6.0–10.6)	(4.0–5.7)	(4.3–6.1)
**Moderate VI (<6/18–6/60)**					
Number	66	16	82	274	356
Percent	16.4%	18.8%	16.8%	7.0%	8.1%
95% CI	(12.7–21.0)	(11.9–28.5)	(13.4–20.9)	(6.2–8.0)	(7.2–9.2)
**Severe VI (<6/60–3/60)**					
Number	1	3	4	14	18
Percent	0.3%	3.5%	0.8%	0.4%	0.4%
95% CI	(0.03–1.2)	(1.2–9.8)	(0.3–2.2)	(0.2–6.4)	(0.3–0.7)
**Blind (<3/60)**					
Number	10	2	12	59	71
Percent	2.5%	2.4%	2.5%	1.5%	1.6%
95% CI	(1.2–5.0)	(0.6–9.2)	(1.3–4.8)	(1.2–2.0)	(1.2–2.1)
**Total Number (Percent)**	402 (100%)	85 (100%)	487 (100%)	3,894 (100%)	4,381 (100%)

VI, visual impairment.

28 people had visual impairment due to AMD alone (i.e., no other visually impairing pathology found), a prevalence of 0.6% (95% CI, 0.4–1.0) for visual impairment from AMD in the population. 9.9% (seven of 71 people) of blindness in this survey was attributable to AMD.

## Discussion

The prevalences of early and late AMD in this African population over 50 y of age were 11.2% and 1.2%, respectively.

Very few data exist on the prevalence and causes of AMD in Africa, and to our knowledge, this is the only population-based study in Africa using an internationally recognised grading system and digital retinal photographs. Although data from Rapid Assessment of Avoidable Blindness surveys exist, these cluster AMD with other posterior segment eye diseases, and so no direct comparisons can be made with the findings from this study. A Nigerian survey used similar methodology, including a population-based approach and fundus photographs; however, retinal imaging was performed only in individuals with a visual acuity of ≤6/12 [26]. In the present study, 75.1% of individuals identified as having AMD had an acuity of 6/12 or greater.

Only one other prospective study of AMD in Africa was found in the peer-reviewed literature. A hospital-based study in South Africa that looked specifically at AMD in Africans was published in 1978 [31]. The study participants were aged 50 y and older, as in this study, and were examined by indirect ophthalmoscopy as well as photography. Higher levels of “senile macular degeneration”, as AMD was then termed, were reported than in this study, affecting 17.4% of participants in the study. It is likely that the hospital population sampled was not representative of the general population, as enrolled participants had attended the hospital eye clinic of their own volition and therefore were more likely to have had symptomatic vision loss than the general population. Typically, the demographic of individuals attending a hospital clinic also differs from the general population in terms of SES and life expectancy. No population-based studies specifically reporting AMD in Africa have been published, to our knowledge.

The prevalence of AMD in this study was also lower than that in a study in the Caribbean, where a 28.7% prevalence of AMD was found [32]. In general, the prevalences in our study are similar to or higher than those documented for Hispanic and Asian populations (range 7.1%–13.6%) [13],[33]–[36], but lower than those found in white populations (range 9.3%–43.1%) [36]–[39].

Direct comparison between studies is not appropriate because of the different grading systems and diagnostic techniques used.

A strength of this study was the high response rate (88.1%). Despite the sophisticated equipment used in the examinations, people were not transported to a fixed examination site, but instead the examination site moved from cluster to cluster. A large, representative population-based sample was examined by SLB and retinal imaging. Another strength of the study is that the same experienced ophthalmologist (W. M.) was present throughout the study and examined all participants. However, a lack of stable electricity supply resulted in the number of people who had retinal images being reduced in some clusters.

A limitation of the study was our not having been able to obtain retinal images for all study participants. This is in large part due to the logistical constraints of performing electricity-dependent examinations in a setting where electricity supply cannot be guaranteed. Univariable analyses comparing those with gradable images (*n* = 3,304) and those without (*n* = 83) found significant differences. Those with no gradable images were more likely to be older, have poor vision, and have a cataract, thus the prevalence for this population may be slightly underestimated. The difference between the groups is likely due to a lack of stable electricity supply in the more rural clusters, where participants were demographically different.

When disease estimates using both methods were compared, SLB was found to have consistently under-diagnosed AMD. An analysis of the false negatives for late AMD revealed that lesions were noted but were not called neovascular AMD; instead they were called macular scars. This may be a reflection of general practice in Africa, where it is often repeated in residency courses that “wet AMD” does not occur in Africans, as has been asserted in several studies [40],[41]. This leads to late AMD being placed far down in the differential diagnosis for macular scars.

There was also discrepancy in identifying pigmentary lesions of early AMD. Retinal imaging identified many more hyperpigmented changes at the macula than did SLB. There are large natural variations in retinal pigmentation, resulting in colour differences between individuals. Such variation can tend to mask the more subtle variation between the important lesion types [42]. Studies have shown that the macular pigment density in other population groups is significantly lower than in African individuals [43]. The study ophthalmologist's perception of what constitutes increased pigment and what is normal background pigment in an African eye, in comparison to the reading centre's criteria, may have led to differences in classification.

Vision is measured based on the person's better eye, whereas AMD affects both eyes, and therefore a disparity between late AMD and poor vision can be seen. For example, there were 38 participants in the present study with late AMD, but only 16 with AMD and a visual acuity <6/60.

Disease subgroups included limited sample sizes, particularly for advanced AMD and blindness, which should be noted when interpreting the results.

Data collection began in 2007 and was based on electoral roll data from 2006. The population demographic is likely to have changed in the time to publication, and given population growth and increased survival, the estimated national prevalence of AMD could underestimate current prevalence.

Of note, location information collected from participants in this study will allow for incidence studies to be carried out in the future, thus providing new insights into the natural progression and incidence of AMD in this population.

### Conclusion

Despite the long held belief that AMD is not a public health concern in Africa, this study provides evidence not only that is AMD as prevalent as in some other world regions (12.4% in this population), but also that it is an important problem contributing to both visual impairment and blindness in Africa. A total of 9.9% of blindness in this survey was attributable to AMD.

New therapeutic strategies have increased the available treatment options and improved prognostic perspectives for AMD in low-income countries [44]. However, these emerging treatments for AMD are largely unavailable in Kenya and most of Africa. When they become available, cost may be a major barrier towards accessing the treatment. Recent evidence suggests that bevacizumab is both effective and relatively affordable [45]–[47], but the infrastructure required to deliver an adequate AMD service, including the use of expensive optical coherence tomography machines, may be prohibitive. It is estimated that over 12 million people in Africa have low vision [48], and AMD is certainly a major contributor. Low vision services remain a hugely neglected area of care on the African continent; strengthening these services might be a cost-effective use of limited resources in the interim period. There is a need to train African-based ophthalmologists to improve recognition and treatment of AMD, particularly neovascular AMD, and a need for research to support the development of treatment programmes that are affordable and deliverable in Africa.

## Supporting Information

Table S1 Comparison of those with diagnosis based on retinal images with those who had only SLB diagnosis.
(DOCX)

Table S2 Demographic characteristics including age as a continuous variable.
(DOCX)

## Abbreviations

AMD: age-related macular degeneration
SES: socioeconomic status
SLB: slit lamp biomicroscopy
