# Supplementary material for: Prevalence of Age-Related Macular Degeneration in Nakuru, Kenya: A Cross-Sectional Population-Based Study
Source: PLoS Med. 2013 Feb 19;10(2):e1001393. doi: 10.1371/journal.pmed.1001393 (PMC3576379; doi:10.1371/journal.pmed.1001393)
Supplement: Table S1 — Comparison of those with diagnosis based on retinal images with those who had only SLB diagnosis. (DOCX) [file pmed.1001393.s001.docx]

###### Table S1

###### Comparison of those with diagnosis based on images with those who only had slit lamp diagnosis

| Attribute | Those with diagnosis done by retinal images  N=3304(%) | Only slit lamp dagnoses  N= 1038(%) | Age and sex adjusted  OR(95%CI) | Univariate p- value | Multivariate adjusted p-value |
| --- | --- | --- | --- | --- | --- |
| Gender |  |  |  |  |  |
| Men | 1629(49%) | 450(43%) | Baseline | P=0.01 | P=0.002 |
| Female | 1675(51%) | 588(57%) | 0.8(0.7-0.9) |  |  |
| Age |  |  |  | P<0.0001 | P=0.06 |
| < 70 years | 2505 (76%) | 718(69%) | Baseline |  |  |
| ≥ 70 years | 799 (24%) | 320(31%) | 0.7(0.6-0.8) |  |  |
| Habitat |  |  |  | P<0.0001 | P<0.0001 |
| Rural | 2143(69%) | 774(75%) | Baseline |  |  |
| Urban | 1161(31%) | 264(25%) | 1.5(1.3-1.7) |  |  |
| SES |  |  |  |  |  |
| Poorest | 783(24%) | 287 (27%) | Baseline |  |  |
| 2^nd^ quartile | 815 (25. %) | 267 (26%) | 1.0(0.9-1.2) | P=0.70 | P=0.8 |
| 3^rd^ quartile | 840 (26%) | 243(23%) | 1.1(0.9-1.4) | P=0.19 | P=0.8 |
| Least poor | 829(25%) | 252 (24%) | 1.1(0.9-1.3) | P=0.58 | P=0.02 |
| Tribe |  |  |  | P<0.0001 | P<0.0001 |
| Kikuyu | 1997(60%) | 721(70%) | Baseline |  |  |
| Kalenjin | 780(24%) | 211(20%) | 1.3(1.1-1.6) |  |  |
| Others | 527(16%) | 106(10%) | 1.7(1.3-2.1) |  |  |
| Diabetes |  |  |  | P=0.004 | 0=0.05 |
| Non diabetic  Diabetic | 3091 (94.1%)  192 (5.9%) | 947 (92%)  86(8%) | Baseline  0.7(0.5-0.9) |  |  |
|  |  |  |  |  |  |
| Visual Impairment |  |  |  | P<0.0001 | P<0.0001 |
| ≥6/12 | 2891(88%) | 820(79%) | Baseline |  |  |
| <6/12 | 3973(12%) | 218(21%) | 0.5(0.4-0.6) |  |  |
| Cataract |  |  |  | P=0.8 | P=0.13 |
| Lens clear | 1877(57%) | 551(54%) | Baseline |  |  |
| Lens opacity | 1406(43%) | 475(46%) | 1.0(0.8-1.2) |  |  |
