# Supplementary material for: Prevalence of Age-Related Macular Degeneration in Nakuru, Kenya: A Cross-Sectional Population-Based Study
Source: PLoS Med. 2013 Feb 19;10(2):e1001393. doi: 10.1371/journal.pmed.1001393 (PMC3576379; doi:10.1371/journal.pmed.1001393)
Supplement: Table S2 — Demographic characteristics including age as a continuous variable. (DOCX) [file pmed.1001393.s002.docx]

Table S2: Demographic characteristics

| Attribute | Those with diagnosis done by retinal images  N=3304(%) | Only slit lamp diagnoses  N= 1038(%) | Age and sex adjusted  OR(95%CI) |
| --- | --- | --- | --- |
| Gender  Men  Female | 1629(49%)  1675(51%) | 450(43%)  588(57%) | Baseline  0.8(0.7-0.9) |
| Age | 3304(100%)  Mean age 63 (62.6- 63.3) | 1038 (100%)  Mean age 64.8 (64.1- 65.5) | 0.98(0.97-0.99) |
| Habitat  Rural  Urban | 2143(69%)  1161(31%) | 774(75%)  264(25%) | Baseline  1.5(1.3-1.7) |
| SES  Poorest  2^nd^ quartile  3^rd^ quartile  Least poor | 783(24%)  815 (25. %)  840 (26%)  829(25%) | 287 (27%)  267 (26%)  243(23%)  252 (24%) | Baseline  1.0(0.9-1.2)  1.0(0.8-1.3)  1.0(0.8-1.2) |
| Tribe  Kikuyu  Kalenjin  Others | 1997(60%)  780(24%)  527(16%) | 721(70%)  211(20%)  106(10%) | Baseline  1.3(1.1-1.6)  1.6(1.3-2.1) |
| Diabetes  Non diabetic  Diabetic | 3091 (94%)  192 (6%) | 947 (92%)  86(8%) | Baseline  0.7(0.6-0.9) |
| Visual Impairment  ≥6/12  <6/12 | 2891(88%)  3973(12%) | 820(79%)  218(21%) | Baseline  0.6(0.5-0.8) |
